# The innovative viscoelastic CP ESP cervical disk prosthesis with six degrees of freedom: biomechanical concepts, development program and preliminary clinical experience

**DOI:** 10.1007/s00590-015-1695-1

**Published:** 2015-09-04

**Authors:** Jean-yves Lazennec, Alain Aaron, Olivier Ricart, Jean Patrick Rakover

**Affiliations:** Pitié Salpétrière Hospital, UPMC, Paris, France; FHI, Quimper, France; Kirchberg Hospital, Luxembourg, Luxembourg; Clinique du Pré, Le Mans, France

**Keywords:** Cervical disk prosthesis, Artificial disk, Viscoelastic disk replacement, Cervical spine mobility, CP ESP, Rotation center, Degenerative cervical disk

## Abstract

The viscoelastic cervical disk prosthesis ESP is an innovative one-piece deformable but cohesive interbody spacer. It is an evolution of the LP ESP lumbar disk implanted since 2006. CP ESP provides six full degrees of freedom about the three axes including shock absorbtion. The prosthesis geometry allows limited rotation and translation with resistance to motion (elastic return property) aimed at avoiding overload of the posterior facets. The rotation center can vary freely during motion. The concept of the ESP prosthesis is fundamentally different from that of the devices currently used in the cervical spine. The originality of the concept of the ESP^®^ prosthesis led to innovative and intense testing to validate the adhesion of the viscoelastic component of the disk on the titanium endplates and to assess the mechanical properties of the PCU cushion. The preliminary clinical and radiological results with 2-year follow-up are encouraging for pain, function and kinematic behavior (range of motion and evolution of the mean centers of rotation). In this series, we did not observe device-related specific complications, misalignment, instability or ossifications. Additional studies and longer patient follow-up are needed to assess long-term reliability of this innovative implant.

## Introduction

Anterior cervical discectomy and fusion (ACDF) is a proven intervention for patients with radiculopathy and myelopathy. A major concern related to the treatment of cervical degenerative disk disease and spondylosis with ACDF is the issue of adjacent segment degeneration [1].

Radiographic evidence of adjacent-level disease (ALD) has been reported to occur in as many as 92 % of patients at 5-year follow-up [2]. Hilibrand et al. [3] calculated a 2.9 % annual risk of symptomatic ALD; survivorship analysis projected that a 25.6 % of the patients who underwent an ACDF would develop symptomatic ALD within 10 years after. There is clinical evidence to support the postsurgical nature of adjacent segment disease. Goffin et al. [2] were able to demonstrate a similar rate of ALD in younger patients with trauma compared with older patients with degeneration following anterior cervical arthrodesis.

The concept that adjacent levels need to compensate for loss of motion in the fused segment is supported by biomechanical studies. Cadaveric testing has demonstrated the finding that arthrodesis generally results in increased adjacent-level intradiscal pressures [4], and statistically significant changes in adjacent-level motion compared with arthroplasty under the same experimental conditions [5]. Studies additionally reveal that the degree of lordosis achieved during fusion significantly alters adjacent-level range of motion [6] and that in some cases, segmental mobility may be maximally increased at levels distant from the index level [7]. In addition, kyphotic deformity following ACDF has been implicated in the development of segmental instability, clinically significant ALD and poor functional recovery. It has been speculated that fusion in kyphosis increases posterior slipping forces onto adjacent vertebral levels and may cause higher loads on the posterior column than a fusion in lordosis [8].

Cervical disk arthroplasty cannot systematically supplant arthrodesis [9], but it has emerged as a promising alternative to fusion in appropriately selected patients [10] to reduce or eliminate ALD by preserving motion at the treated level.

The primary goals of the procedure are to preserve or to restore normal spinal kinematics. Nevertheless, although the range of motion (ROM) is an important feature of an artificial disk, it is only a single aspect of spinal biomechanics: elastic resistance to movement, twisting potential and elastic resistance to load bearing are major properties of the anatomical disk. Normal kinematics should not be analyzed only as a movement on the three planes, but also as elastic resistance to dynamic stress on these three planes.

The aim of motion preservation is also to neutralize excessive movements while preserving the physiologic biomechanical properties of the functional spinal unit (FSU) involved to interrupt the progression of degenerative processes and to prevent ALD. Elastic resistance of the FSU is a biomechanical property often underestimated but crucial for the stability of the spine. The biomechanics of cervical implants takes into account only the ROM of the devices and not the elastic resistance: the risk is a greater ROM in comparison with a normal disk, especially in rotation, with a potential overloading of the facet joints [11].

In terms of the quality of vertebral motion, the instantaneous center of rotation (ICR) during flexion–extension is considered as a major parameter. The ICR location depends on the cervical FSU level. In addition, Liu et al. described a correlation with the age-related degeneration [12]. Cervical arthroplasty should be optimized or adaptive enough for attempting an ICR location close to physiologic kinematic conditions of the motion segment according to physiologic aging, normal degenerative changes and local potential evolution.

The effect of this motion-sparing alternative on angulation at the treated level and on the overall spinal alignment may be important to long-term clinical outcomes and rates of adjacent segment disease. The control of stability and ROM is critical to optimize and maintain local and regional balance.

In addition, the impact on axial loading and shock absorption of the FSU needs to be addressed [13]. Successful reproduction of physiologic kinematics and long-term viability of the disk replacement must consider multiple factors, including facet loading, disk height, device design and biomaterials, and implant position [14]. The goal is to obtain the potential to approximate the axes of rotation of the native segment in flexion–extension, lateral bending and axial rotation, and a graded resistance to motion.

Total disk arthroplasty devices can be classified according to modular versus nonmodular design, fixation properties, articular design and composition (uniarticular, biarticular and nonarticular), and kinematics (constrained, semiconstrained and unconstrained) [15, 16].

The viscoelastic cervical disk prosthesis ESP is an innovative one-piece deformable but cohesive interbody spacer. It is an evolution of the LP ESP lumbar disk implanted since 2006 [17].

Recognizing that the human disk does not work like a joint but as a “silent bloc,” this technology meets a critical need: in the cervical spine, the shock absorption and the control of stability are very important to avoid side-level degeneration, hypermobility and rotational or sagittal imbalance. In addition, the biomechanical constrains are very different from a disk level to each other as far as mobility is concerned. Moreover, one-third of the asymptomatic population has cervical kyphosis [18], and recent literature has pointed out significant modifications of the ICR according to the age and the degree of cervical degeneration [12]. The complexity of these anatomical and functional data shows the limitations of conventional mechanical prostheses and highlights the potential interest of viscoelastic concept for cervical prostheses.

CP ESP provides six full degrees of freedom about the three axes including shock absorption.

The prosthesis geometry allows limited rotation and translation with resistance to motion (elastic return property) aimed at avoiding overload of the posterior facets. The rotation center can vary freely during motion. It thus differs substantially from current prostheses.

The goal of this paper is to present this innovative concept and the preliminary clinical results and radiological outcomes. This study reports the results of a prospective pilot evaluation of the first patients implanted since 2012. In addition to measuring ROM, we were specifically interested in the quality of the kinematics and thus we investigated the mean center of rotation at both the instrumented and adjacent levels.

## The implant

Because the healthy human intervertebral disk has a deformable elastic structure with six degrees of freedom, elastomeric one-piece intervertebral prostheses might be the most physiologic implant for mimicking physiologic levels of shock absorption and flexural stiffness. The ESP^®^ concept (currently lumbar and cervical disk prostheses marketed as the elastic spine pad) was in development for 20 years. With the technological advancements of the ESP^®^, the problem of how to bond the elastic component and the titanium endplates of the disk is solved. Following promising in vitro and in vivo testing, the LP ESP gained clinical approval for use in Europe in 2005 for the lumbar levels [19]. More recently, the cervical version has been CE approved in 2012 for the first implantations.

**Fig. 1:**
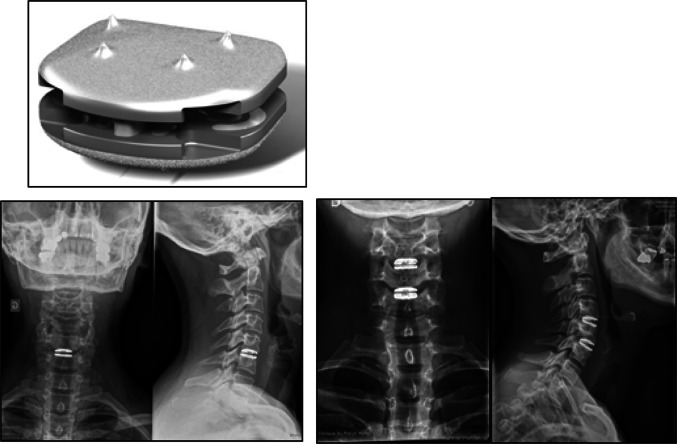
Radiological images with CP ESP disk implant

The design of the CP ESP^®^ prosthesis is based on the principle of the silent block bush (Fig. 1). The CP ESP^®^ is a one-piece deformable implant including a central core made of polycarbonate urethane (PCU) securely fixed to titanium endplates. The endplates have anchoring pegs to provide primary fixation and are covered by a textured T 40 titanium layer and hydroxyapatite to improve bone ongrowth. This center cushion is bonded to the titanium alloy endplates via adhesion molding and employs a peripheral groove without using glue for reinforcement. This type of fixation prevents fluid infiltration and fatigue fractures of the interface, despite the disparate mechanical properties of the polymer and metal endplates. The shape of the cushion has been studied to obtain an optimization of the mobility as well as the control of translation and shear movements during cervical spine mobility.

**Fig. 2:**
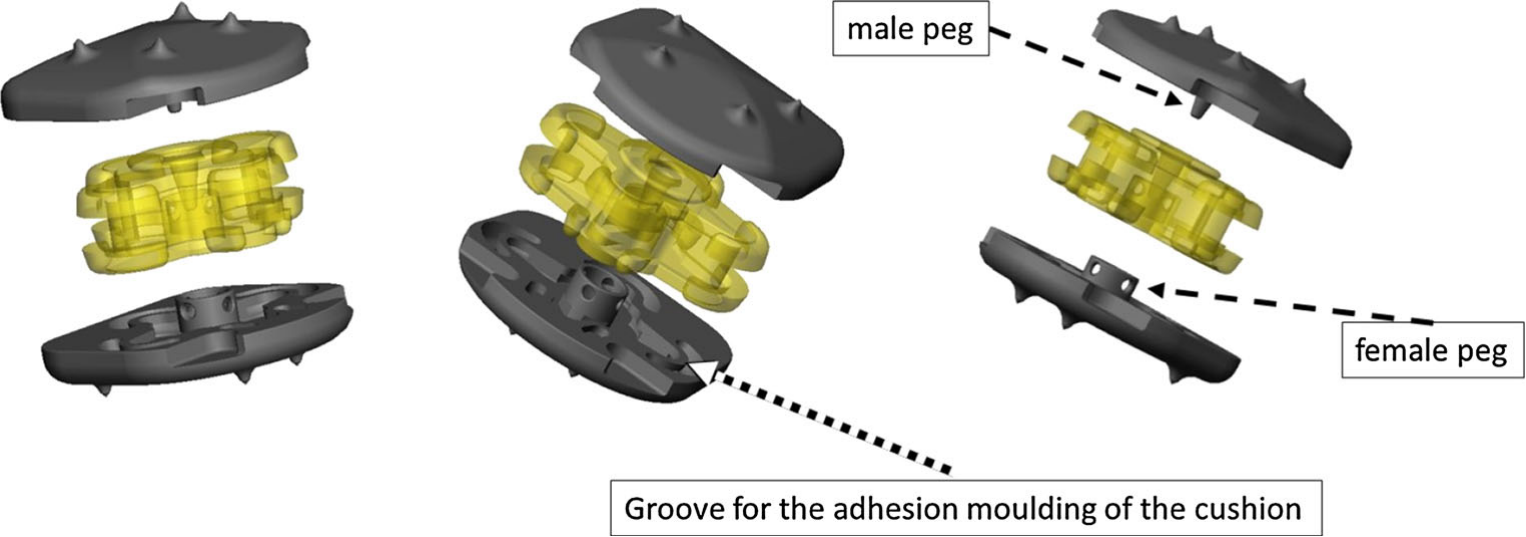
Description of the implant

The PCU annulus is stabilized by supplementary “male” and “female” inner pegs located on the internal surface of both metal endplates. The geometry and position of the pegs, between the peripheral groove and the central area of the endplates, were planned to control compression and translation (Fig. 2). These two pegs, with their contactless fit of the male and female caps, serve to limit shearing during anteroposterior and medio-lateral translation. Through this mechanism, as well as crushing the annulus between the metal plates, the inner pegs also provide cushioning and compressing. These pegs also limit shearing when the endplates are inclined to the horizontal.

The prostheses are available in three thicknesses (5, 6 and 7 mm), each with three sizes in AP and lateral dimensions (13 mm × 15 mm, 14 mm × 17 mm and 15 mm × 20 mm). Regardless of the model, however, the mechanically active cushion and the mechanical properties of the prosthesis are the same: the differences in thickness do not affect the prosthesis’s mobility or its cushioning, even shock-absorbing, effect. The design of the prosthesis allows a range of 14° for flexion–extension, 12° for right-left lateral bending and 8° for rotation. In addition, a 0.8-mm AP translation is possible. Overall, the principle of the CP ESP^®^ makes it possible to reproduce the anisotropy of the healthy disk, and the design allows the control of translation and of return torque.

## Mechanical properties

The CP ESP^®^ prosthesis, with its “silent block bush” system, improves upon fixed centers of rotation present in other, implants with an articulated design [20]. Additionally, for each direction requested, the CP ESP^®^ prosthesis offers resistance that becomes greater with the amplitude of the movement. Thus, the CP ESP^®^ is not directly comparable to the older first-generation implants. As a third-generation prosthesis, it exhibits six degrees of freedom while offering a cushioning effect and restoring elasticity. The mechanical attributes of CP ESP approach those reported in the literature for a normal human disk. Rather than focusing on the global mobility of the implant, the research studies have addressed the reconstitution of the stiffness for the different mobility sectors expressed as bending and rotational moments (Table 1).

**Table 1:** Comparison of the characteristics of the CP ESP implants and normal cervical disk as reported in the literature

	CP ESP	Natural disk
Stiffness for compression	733 N/mm	492 N/mm
Moment for extension	0.03 Nm/1°	0.5 Nm/1°
Moment for flexion	0.03 Nm/1°	0.03 Nm/1°
Moment for lateral bending	0.05 Nm/1°	0.9 Nm/1°
Moment for rotation	0.24 Nm/1°	0.8 Nm/1°

## Biomechanical assessment: fatigue and wear tests

**Fig. 3:**
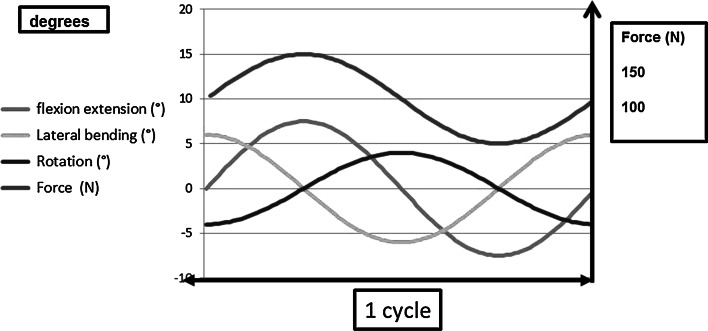
Description of the cycle testing according to ISO 18192 Norm

**Fig. 4:**
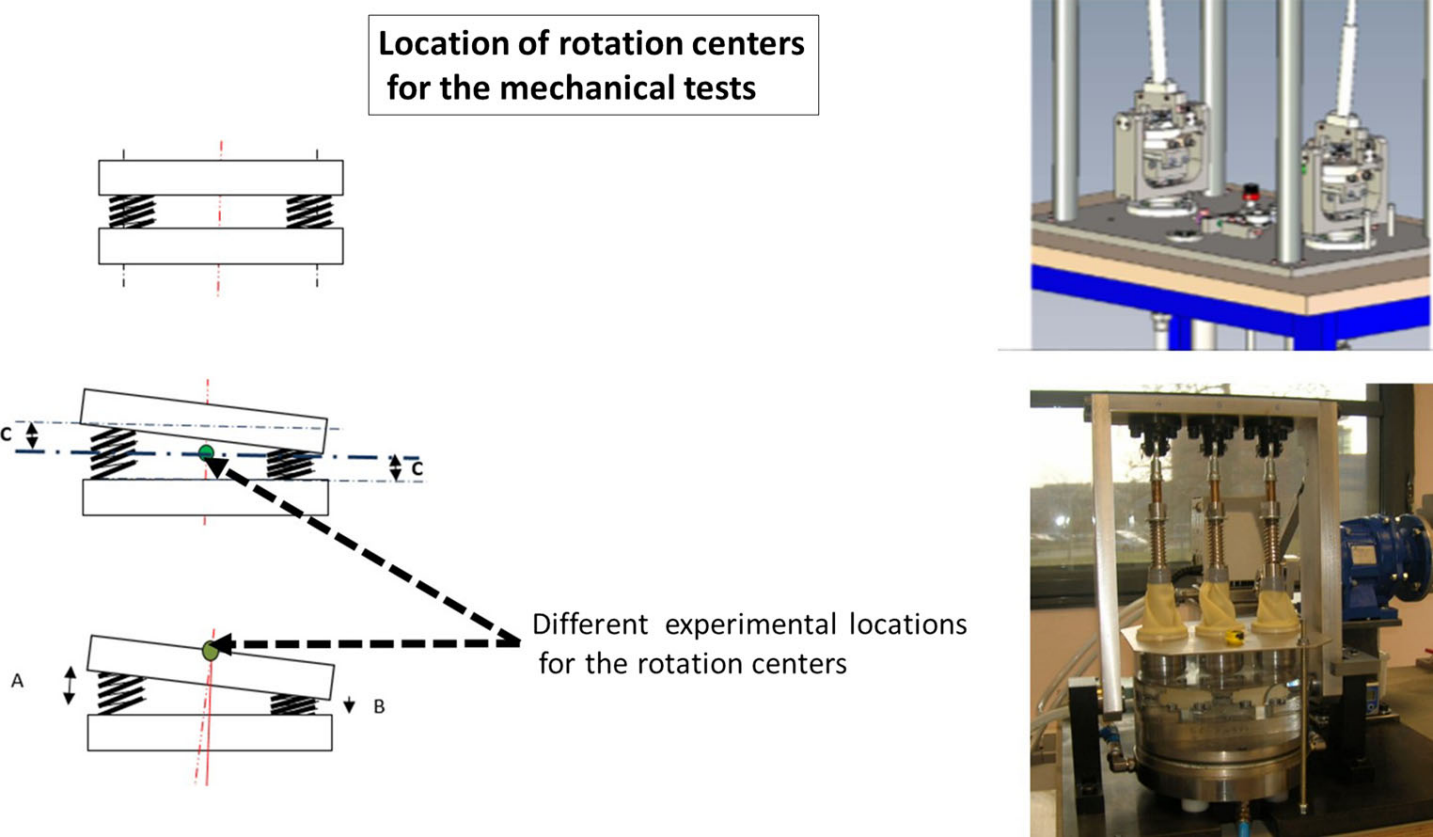
Different configurations have been tested regarding the location of the rotation centers to address the worst case scenarios during the tests

The originality of the concept of the ESP^®^ prosthesis led to innovative and intense testing of various sorts. Wear tests were conducted in a three-axis motion simulator according to the ISO 18192 Norm (Fig. 3). Different configurations have been tested regarding the location of the rotation centers to address the worst case scenarios during the tests (Fig. 4). According to the different protocols, the loss of height ranged from 0.02 to 0.12 mm after 10 million cycles. No particles were detected in the water bath. As a comparison, the literature reports a loss of height of up to 0.02 mm/million cycles for the Bryan disk. For this implant, the amount of particles has been reported as 0.96 mm^3^/million cycles.

The mean loss of weight reported for the Bryan disk has been 6 mg/million cycles. In our testing protocol, the mean variation of weight has been 0.8 mg for 10 million cycles for the CP ESP. Comparison with other implants is reported in Table 2. In addition, the alignment of the superior and inferior endplates has been studied after these series of tests to assess the cohesion of the cushion and metal parts. After 10 million cycles, the variation was insignificant (0, 2 mm for AP and lateral measurements).

**Table 2:** Literature summary of biomechanical simulations for wear

References	Device	Simulation	Mass loss	Height loss	Volume loss
Anderson et al. [21]	Bryan	6 disks: 10 million cycles; 150 N force flexion/extension, lateral bending and axial rotation in 37 °C calf serum sample; bending + 10 million cycles rotation at 4 Hz; 4 disks: tested until and plate to endplate contact	1.75 % (after 20 million cycles in 6 disks)	0.48 % (after 20 million cycles in 6 disks)	0.57 mm^3^/million cycles
Anderson et al. [21]	Prestige	10 million cycles of 148 N flexion/extension, 5 million cycles of 49 N lateral bending and 5 million cycles of 49 N axial rotation at 2 Hz in 37 °C calf serum sample; number of disks tested not reported	NR	NR	0.18 mm^3^/million cycles (0, 19 % loss after 20 million cycles)
FDA [22]	ProDisc C	10 million cycles of 150 N flexion/extension, lateral bending and axial rotation at 1 Hz in 37 °C calf serum sample; debris sampled every million cycles	2.59 mg/million cycles	NR	NR
Grupp et al. [23]	Active C	6 disks tested with a ISO 18192-1:2008 (E) process: flexion, extension, lateral bending and axial rotation movements in sinusoidal wave from with 150 N flexion force and 50 N extension force, 10 million cycles at 1 Hz in 37° C calf serum sample	1.0 mg/million cycles	0.03 mg/million cycles for polymer inlay	1.0 mm^3^/million cycles
FDA [24]	PCM	5 disks tested under Draft ASTM F243-05 conditions (±10° fully reversing lateral bending, ±6° fully reversing axial)	Average cumulative wear at 10 million cycles was 71.22 ± 17.56 mg. Wear rate 0.042 mg/million cycles between 3 and 10 million cycles	NR	NR
FDA [25]	Secure-C	150 N constant compressive load for 10 million cycles. Calf serum and deionized water solution with EDTA, maintained at 37 °C Stage 1: 6 disks for 10 million cycles of complex loading at a frequency of 2 Hz using combined flexion/extension (±7°), lateral bending (±7°) and axial rotation (±1.5°) Stage 2: tested with increased axial rotation (±6°)	Stage 1: 2.57 mg ± 1.21 mg per million cycles Stage 2: 0.89 mg ± 0.3 mg per million cycles	NR	NR
FDA [26]	Mobi C	6 disks: 10 million cycles; frequency of 1 Hz Combined flexion/extension (±7.5°), lateral bending (±6°) and rotation (±4°) under axial compression (50–150 N)	1.456 ± 0.075 mg/million cycles	NR	NR
FDA [27]	Prestige LP	Stage 1: 6 disks tested in accordance with ASTM 2423 (lateral bending coupled with axial rotation followed by flexion/extension) Stage 2: 6 disks tested in accordance with ISO 18192-1 (lateral bending combined with axial rotation and flexion/extension)	NR	NR	Stage 1: 0.35 ± 0.03 mm^3^/million cycles total wear at 20 million cycles: 4.22 ± 0.21 mm^3^ Stage 2: steady-state wear rate 0.25 ± 0.04 mm^3^/million cycles total accumulated wear 2.74 ± 0.38 mm^3^
	CP ESP	3-Axis motion simulator according to the ISA 18192 Norm	Mean variation of weight 0.8 mg for 10 million cycles	Loss of height ranged from 0.02 to 0.12 mm after 10 million cycles	

NR indicates not reported

## Structural tests: validation of the final stage of coating on the exterior side of the metal plates

Adding a further final coating of porous titanium and spraying hydroxyapatite on the implant in its permanent form cause its temperature to rise. To ascertain the effect of this temperature increase on the flexible center cushion (at 120 °C, there is a known risk of PCU deterioration), tests were conducted. The tests verified that the temperature rise did not affect the structure of the PCU.

## Biostability tests

To evaluate the biostability of the ESP after conducting a wear test of 10 million cycles, we used a demineralized water bath and analyzed the particles collected during filtration. This method is consistent with the ISO standard 10993-13/biological evaluation of medical devices, Part 13: Identification and quantification of the decay products of polymer-based medical devices. We used a scanning electron microscope (SEM LEO I455VP), equipped with an energy-selective spectrometer (EDS OXFORD). No particles from the component materials of the prosthesis were found. These results were consistent with previous studies on the LP ESP disk.

## PCU aging test

The specific PCU used for the LP ESP prosthesis is not oxidized during storage (bionate 80A (DSNM Biomedical, The Netherlands) according to master file MAF844) [28].

To evaluate this claim, the PCU was artificially aged in 80 °C water, in accordance with recommendations for aging plastics such as the UHMWPE (ASTM standard F 2003: Accelerated aging of ultra-high molecular weight polyethylene after gamma irradiation in air). The aged PCU was then subjected to compression loads from 150 N to 1250 N, consistent with ISO standard 18192 (intervertebral spinal disk prostheses Part 1: Loading and displacement parameters for wear testing and corresponding environmental conditions for test) for wear tests, for 10 million cycles.

No modification of the Fournier transform infrared spectrum or any modification of the mean molecular weight (ASTM standard D 5296) was observed. The PCU aging test results were comparable to those reported in the literature [29].

## Biocompatibility tests

Biomatech (Chasse-sur-Rhone, France) conducted the biocompatibility tests (Table 3). The material components were evaluated individually and as a finished assembly, as specified in ISO standard 10993 (Biological Evaluation of Medical Devices): Cytotoxicity was assessed using ISO standard 10993-5. Testing for sensitization conformed to ISO standard 10993-10.

**Table 3:** Biocompatibility tests for CP ESP

Tests	Results	Standard
Mutagenicity	Nonmutagenic	OECD N° 471
Chronic toxicity ESP muscle implantation	Macroscopic reaction nonsignificant	ISO 10993-1
Hemolysis	Nonhemolytic	ISO 10993-4
Humoral immunological study	No humoral (serological) immune response	OECD N° 407
ESP pyrogenicity	Nonpyrogenic	ISO 10993-11
Sensitization	No dermal sensitization	ISO 10993-10
Acute systemic toxicity	No significant systemic toxicity	ISO 10993-11
ESP implantation 7 days in rabbits	Macroscopic reaction not significant	ISO 10993-6
Intracutaneous toxicity	No significant toxicity or irritation	ISO 10993-10
Carcinogenicity 2 years in rats	Noncarcinogenic	ISO 10993-3

Employing ISO standard 10993-10, the components and assembly were tested for irritation or intradermal reaction. In addition, acute systemic toxicity was examined using ISO standard 10993-11 Chromosomal genotoxicity (Hearts test), and chromosomal anomalies were tested in accordance with ISO standard I 0993-3.

The Biomatech biocompatibility testing revealed that the ESP devices also satisfy the criteria of the FDA’s subacute sensitization test (FDA—Guidelines for Toxicity Tests Chapter IV).

## Clinical study

Our evaluation program was organized in two stages:

- a preliminary study with 1-year follow-up to detect potential dysfunctions and early postoperative stabilization problems or adjustment difficulties
- a 2-year analysis focused on radiological progression in terms of mobility and centers of rotation of analysis

### The cases

We analyzed prospectively 62 consecutive cases included in a preliminary study according to the classical indications and contraindications for cervical disk replacement. The aim of this work is to provide a snapshot of the evolution of our series since the first implantations.

The surgeries were performed by two senior surgeons. There were 39 women and 23 men in this group. The average age was 45 ± 8 years (29–60). The implantation was on a single level in 74 % of the CP ESP and on two levels in 26 %. Globally, 71 CP ESP prostheses were analyzed (Table 4).

**Table 4:** Description of the pilot study

	Operated levels	Patients	CP ESP implants
1 level	C3C4	1	53 (74 % of the implants)
	C4C5	4	
	C5C6	26	
	C6C7	20	
	C7D1	2	
2 levels	C4C5/C5C6	2	18 (26 % of the implants)
	C5C6/C6C7	6	
	C6C7/C7D1	1	
			71 implants

Clinical data and X-rays were collected at the preoperative time and at 3, 6, 12 and 24 months post-op.

The functional results were measured using Neck and Arm VAS, NDI, SF-36 (physical component PCS and mental component MCS). The analysis was performed by a single observer who was independent from the selection of patients and from the surgical procedure.

### One-year follow-up results

The mean operative time was 48 min (40–75 min) (SD 9 min). The hospital stay was 3.2 days (SD 0.8) (1–5 days).

We did not observe device-related specific complications. In this series, we did not face misalignment, instability or ossifications.

All clinical outcomes significantly improved at every time points when compared to the preoperative status (Table 5). Table 6 summarizes the changes in the variations of ROM over time.

**Table 5:** Clinical outcomes of the preliminary study

Mean ± SD	Pre-op	3 Months	6 Months	12 Months
(*a*)				
VAS neck (/10)	6	2.65	1.74	2.65
VAS arm (/10)	6.3	2.7	1.8	2.4
NDI (%)	56 ± 16	32.7 ± 17	22.1 ± 16	24 ± 17
NDI points	27.4 ± 8.8	16.2 ± 8.6	10.9 ± 7.9	11.9 ± 8.5
SF-36 PCS score	31	48	61	56
SF-36 MCS score	32	50	63	62

		Pre-op/3 Months	Pre-op/6 Months	Pre-op/12 Months
(*b*) *Mann–Whitney test for the evolution of the clinical tests (VAS, NDI, SF-36 P, SF-36* *M)*				
VAS neck	Conclusion			
	*P* value	0.01	0.01	0.01
VAS arm	Conclusion			
	*P* value	0.01	0.01	0.01
NDI	Conclusion			
	*P* value	0.01	0.01	0.01
SF-36 PCS	Conclusion			
	*P* value	0.01	0.01	0.01
SF-36 MCS	Conclusion			
	*P* value	0.01	0.01	0.01

**Table 6:** Evolution of the ROM of the implanted and adjacent levels

Degrees (°)	3 Months	6 Months	12 Months
ROM of the instrumented level	6.8 ± 4.1	10.3 ± 5.0	8.4 ± 4.3
ROM of the upper adjacent level	9.7 ± 4.9	11.7 ± 5.5	12.9 ± 6.8
ROM of the lower adjacent level	6 ± 4.1	10 ± 5.0	9.4 ± 5.7

**Fig. 5:**
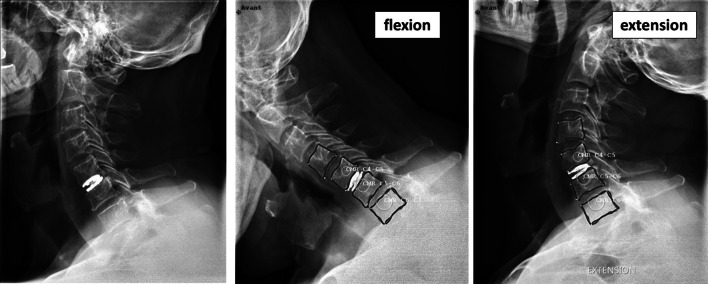
Mean centers of rotation (MCR) at the instrumented and at the upper and lower adjacent levels measured for flexion/extension using Spineview^®^ software; we can observe the adaptation of the implant including a translation for flexion

The mean centers of rotation (MCR) at the instrumented and at the upper and lower adjacent levels were measured in flexion/extension using Spineview^®^ software (3-, 6- and 12-month follow-up) (Fig. 5). Their evolution illustrates the versatility and the forgiveness of the CP ESP implants according to the levels and the various sagittal balance.

### Two-year follow-up results

**Fig. 6:**
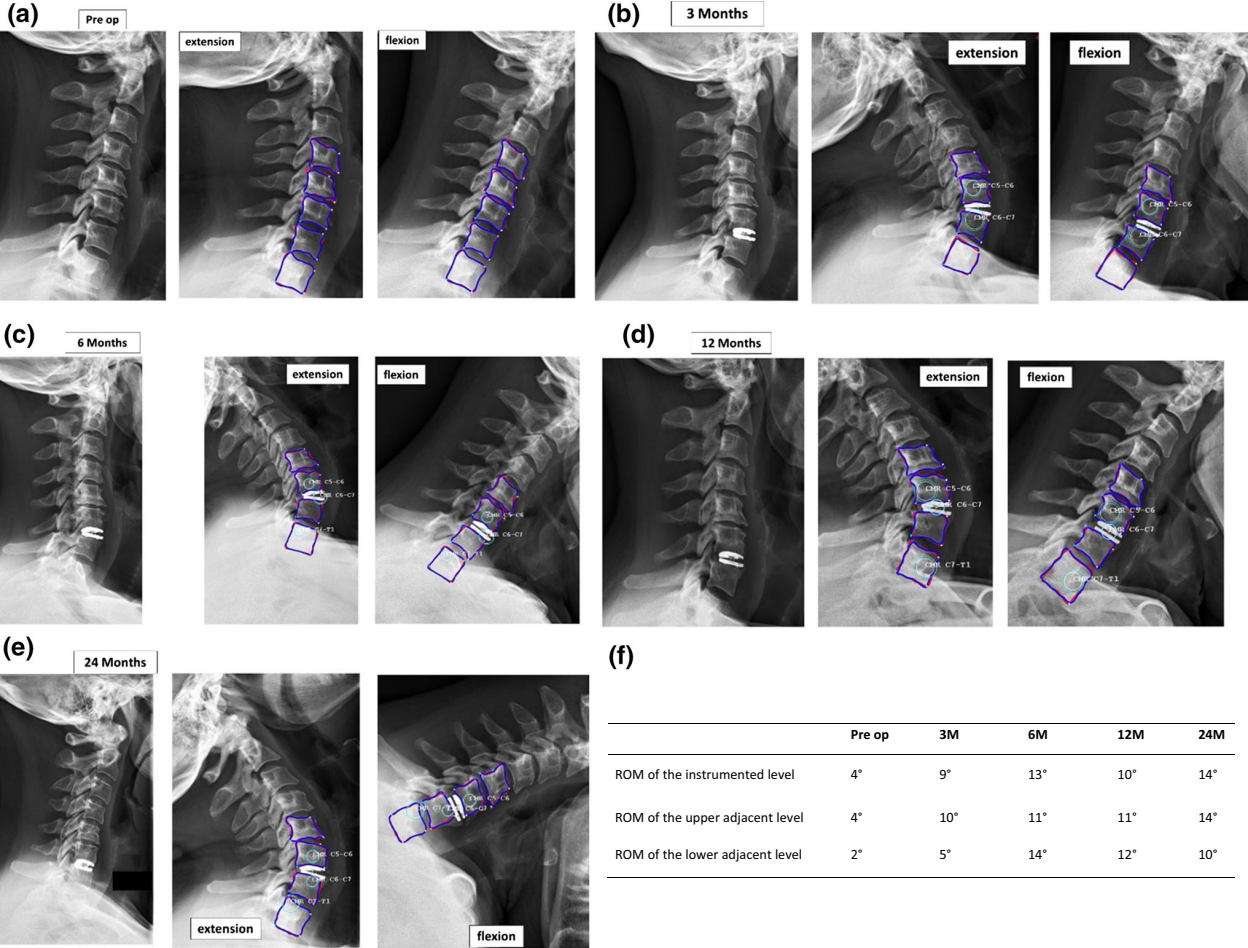
**a**–**f** Mean centers of rotation (MCR) and mobility at the instrumented and at the upper and lower adjacent levels in a patient with global cervical kyphosis: adaptation during the follow-up from 3 to 24 months

The evolution of clinical and radiological parameters confirms the good results already observed at 12 months of follow-up (Table 7; Fig. 6a–f).

**Table 7:** Clinical and radiological outcomes after 2-year follow-up

Mean ± SD	24 Months
VAS neck (/10)	2.9
VAS arm (/10)	1.6
NDI (%)	19 ± 17
NDI points	9.3 ± 8
SF-36 PCS score	64.2
SF-36 MCS score	68.6
ROM of the instrumented level	10.7° ± 4.2°
ROM of the upper adjacent level	13.8° ± 6.5°
ROM of the lower adjacent level	11.1° ± 8.2°

## Conclusion

The design of the CP ESP^®^ prosthesis provides stability by limiting rotation and translation, which prevents overload of the posterior facet joints. At the same time, the ESP center of rotation can fluctuate during motion. While achieving six degrees of freedom including vertical translation, this viscoelastic prosthesis also supplies cushioning, which offers shock absorption. The CP ESP thus distinguishes itself substantially from other current two- or three-piece prostheses that contain l or 2 bearing surfaces and provide only 3 or 5 degrees of freedom. These other devices also offer no or very little resistance and provide no elastic return. This study reports encouraging clinical results about pain, function, kinematic behavior and ROM. We concede that additional clinical studies and patient follow-up are needed to assess long-term reliability. However, the results we describe here suggest the outcomes that surgeons and patients might anticipate.

The optimal ROM after TDR for limiting adjacent segmental disease has not yet been established. The radiological evaluation of the results is classically focused on the ROM, but the quality of movement is also an issue, especially at the cervical levels as coupled motions are a key point. The CP ESP acts as a deformable but cohesive interbody spacer that provides six full degrees of freedom about the three axes. This allows instantaneous axis of rotation change freely, as in the normal disk, which can optimize a functional coherence with the facet joints mechanics. It is also a significant benefit for the adaptation to the various disk levels as the biomechanical constrains are different from C3 to T1. Our preliminary experience did not point out complications as sagittal misalignment, ossifications and instability. An interesting point is the evolution of the MCR in the postoperative course. This adaptation ability is one of the main features of this promising implant as we need to consider the mean and long-term evolution of the global cervical posture and mobility after a cervical disk replacement.
